# Barriers to and facilitators of the provision of a youth-friendly health services programme in rural South Africa

**DOI:** 10.1186/1472-6963-14-259

**Published:** 2014-06-16

**Authors:** Rebecca Sally Geary, Francesc Xavier Gómez-Olivé, Kathleen Kahn, Stephen Tollman, Shane Anthony Norris

**Affiliations:** 1 Department of Infectious Disease Epidemiology, School of Public Health, Imperial College London, London, UK; 2 Department of Population Health, Faculty of Epidemiology and Population Health, London School of Hygiene and Tropical Medicine, London, UK; 3 MRC/Wits Rural Public Health and Health Transitions Research Unit (Agincourt), School of Public Health, Faculty of Health Sciences, University of the Witwatersrand, Johannesburg, South Africa; 4 Umeå Centre for Global Health Research, Department of Public Health and Clinical Medicine, Umeå University, Umeå, Sweden; 5 INDEPTH Network, Accra, Ghana; 6 MRC/Wits Developmental Pathways for Health Research Unit, Department of Pediatrics, Faculty of Health Sciences, University of the Witwatersrand, Johannesburg, South Africa

**Keywords:** Reproductive Health Services, Adolescent, Youth, South Africa, Rural Health Services, Adolescent Health Services, Youth friendly

## Abstract

**Background:**

Youth-friendly health services are a key strategy for improving young people’s health. This is the first study investigating provision of the Youth Friendly Services programme in South Africa since the national Department of Health took over its management in 2006. In a rural area of South Africa, we aimed to describe the characteristics of the publicly-funded primary healthcare facilities, investigate the proportion of facilities that provided the Youth Friendly Services programme and examine healthcare workers’ perceived barriers to and facilitators of the provision of youth-friendly health services.

**Methods:**

Semi-structured interviews were conducted with nurses of all eight publicly-funded primary healthcare facilities in Agincourt sub-district, Mpumalanga Province, South Africa. Thematic analysis of interview transcripts was conducted and data saturation was reached.

**Results:**

Participants largely felt that the Youth Friendly Services programme was not implemented in their primary healthcare facilities, with the exception of one clinic. Barriers to provision reported by nurses were: lack of youth-friendly training among staff and lack of a dedicated space for young people. Four of the eight facilities did not appear to uphold the right of young people aged 12 years and older to access healthcare independently. Breaches in young people’s confidentiality to parents were reported.

**Conclusions:**

Participants reported that provision of the Youth Friendly Services programme is limited in this sub-district, and below the Department of Health’s target that 70% of primary healthcare facilities should provide these services. Whilst a dedicated space for young people is unlikely to be feasible or necessary, all facilities have the potential to be youth-friendly in terms of staff attitudes and actions. Training and on-going support should be provided to facilitate this; the importance of such training is emphasised by staff. More than one member of staff per facility should be trained to allow for staff turnover. As one of a few countrywide, government-run youth-friendly clinic programmes in a low or middle-income country, these results may be of interest to programme managers and policy makers in such settings.

## Background

Youth is commonly thought of as a period of optimum health. However, in sub-Saharan Africa, the prevalence of HIV, other sexually transmitted infections (STIs), and adolescent childbearing among young people is high [1-3]. There are more than a million new HIV infections among people aged 15–24 years worldwide each year, which account for 41% of new infections among those aged 15 years and older [4]. Worldwide, HIV/AIDS is the second leading cause of death among young adults (aged 20–24 years) [5]. Adolescent childbearing is associated with negative health outcomes for both the adolescent mother and the infant [5,6].

In South Africa in 2011, HIV prevalence was 12% among young women (aged 15-24 years) and 5% among young men [7]. Half of women have given birth by the age of 20 years and two thirds of adolescent (15-19 years) pregnancies are reported as unwanted [8]. Nine percent report having had sex before the age of 15 years, and early sexual debut is associated with increased risk of HIV infection, other STIs, adolescent pregnancy, forced sex, and an increased number of lifetime partners as well as with decreased use of condoms and other contraceptives [9-19]. Knowledge about sexuality and reproductive health among young men and young women is limited and young people report a need for more information on relationships, pregnancy and STIs [2,20]. Fear of judgmental attitudes of healthcare workers has been reported as a barrier to young people’s use of a range of health services in South Africa [21-25].

From these statistics it is clear that young people in South Africa have a need for sexual and reproductive health information and services that is not currently being met. Efforts to improve young people’s health should include the provision of youth-friendly primary care services; the International Conference on Population and Development Plan of Action, the Maputo Plan of Action and the World Health Organisation (WHO) have called for the development of these services worldwide, and their provision has been defined as a key goal for reducing the vulnerability of youth to HIV [26-30]. Whilst there are some examples of successful, small-scale youth-friendly services worldwide, these projects often have limited coverage or limited periods of implementation or follow-up [31-33]. To generate significant improvements in young people’s use of health services, and in their sexual and reproductive health, such interventions will need to be scaled up and implemented over longer time frames [34]. This reinforces the need for evidence on the sustainability of youth-friendly health services interventions, on barriers to and facilitators of the scale-up and implementation of these interventions, and their impact on young people’s health and use of health services.

The Youth Friendly Services (YFS) programme in South Africa is one of the few youth-friendly health services interventions to have been scaled-up. The Department of Health (DoH) took over the management of this programme from the non-governmental organisation (NGO) loveLife in 2006. Between 1999 and 2006 loveLife managed this programme under the name of the National Adolescent Friendly Clinic Initiative (NAFCI) as one component of a national HIV prevention campaign which combined a sustained, multi-media HIV awareness and education campaign with outreach services including youth centres (Y-Centres) and peer educators (known as groundBREAKERS) [35]. NAFCI involved training service providers, efforts to improve facilities, multi-media campaigns and activities in the community and with other sectors [36]. A set of “adolescent-friendly” standards that included those relating to the types of services provided, policies supporting adolescents’ rights to healthcare and the clinic environment were defined for clinics to work towards using a facilitated approach [37]. The DoH was an active partner from the programme’s inception, and by 2005, 350 clinics nationwide were involved [38].

In 2006, the DoH agreed to take over the management of a simplified version of NAFCI, comprising training healthcare providers and facility accreditation, under their Youth Friendly Services (YFS) programme [38,39]. The National Department of Health and key stakeholders, including the NGO loveLife, defined a core package of services for the Youth Friendly Services programme (to be implemented in primary healthcare facilities) that aim to improve the sexual and reproductive health of both young men and young women. YFS’s target group is young people aged 10-24 years and it aims to: promote access and utilisation of YFS, improve the health status of young people, build the capacity of health care providers to provide YFS and to promote services for HIV-infected and HIV-exposed young people. The “adolescent-friendly” standards defined for NAFCI remain integral to YFS [40]. LoveLife supports the DoH by developing training curricula, programme guidelines and implementation tools, and by facilitating YFS training for DoH practitioners at the Department’s request [41]. DoH figures indicate that in 2010/11, 47% of publicly-funded primary healthcare facilities in South Africa were implementing YFS [42]. The DoH aims to have 70% of primary healthcare facilities implementing this programme by 2012/13 [43].

Earlier work identified the YFS programme as an effective approach for implementing a youth-friendly clinic programme within a public health system in terms of pre-defined standards that included: the types of services provided, the clinic environment and policies supporting adolescents’ rights [38]. However, previous evaluations did not investigate the barriers to and facilitators of its implementation experienced by healthcare workers, and no evaluations have been published since the South African DoH took over the programme’s management [44-47]. In the context of the programme’s handover to the DoH in 2006, and high coverage targets, it is timely to investigate both current provision and healthcare workers’ perceptions of barriers to and facilitators of YFS provision.

We aimed to investigate provision of youth-friendly health services in a rural former “homeland” (part of the Bantustan system during *apartheid*) in South Africa with high adolescent fertility and HIV-prevalence [48,49]. Objectives were, first, to describe the services provided at each of the eight health facilities in this sub-district, including whether the Youth Friendly Services programme was provided, and secondly, to examine barriers to and facilitators of the provision of youth-friendly health services as perceived by healthcare workers. Questions involving young people’s perceptions and experiences of the programme will be investigated in further work. This study focused on formative questions relevant to sustained provision of health information and services for young people in this and similar rural settings.

This study was conducted in 2011 in the Agincourt sub-district of Bushbuckridge, Mpumalanga Province, South Africa, which borders the Kruger National Park and southern Mozambique. In 2010 Mpumalanga Province had the second highest provincial HIV prevalence among antenatal care attendees in South Africa at 35.1% [48]. While fertility in other age groups in Agincourt has declined, adolescent fertility has remained relatively high [49]. The Agincourt sub-district covers approximately 420 km^2^, with some 90,000 people living in 27 villages under both traditional and civic leadership [50]. Physical infrastructure is limited; there is no formal sanitation system, piped water to communal standpipes is erratic and electricity is unaffordable for many. All villages have a primary school and attendance is almost universal. There are several high schools, but half of 20 year olds are still enrolled indicating lagging academic progress. High unemployment contributes to male and female temporary labour migration [50]. The study site has been described in detail elsewhere [50-52]. Health and demographic surveillance was introduced in 1992 and the study area has a strong record of health systems research and development [51,53,54].

## Methods

Seven publicly-funded primary healthcare clinics and a larger health centre are located within the Agincourt sub-district. At each of these sub-district health facilities a professional nurse, most commonly the nurse-in-charge, was invited to participate in the study. Semi-structured interviews were conducted in English and a local fieldworker attended the interviews to assist with introductions and any communication difficulties between English and Shangaan. Interview questions were pre-defined to address the aims of the study and covered the following topics at each health facility: the services available to young people, opening hours, confidentiality, perceived community support for the provision of health services to young people, provision of the Youth Friendly Services programme or other activities related to youth-friendly health services and reflections on providing health services to young people.

Seven of the eight interviews were audio-recorded and the interviewer transcribed recordings verbatim. A number of broad themes for the analysis were pre-defined based on formative questions relevant to the design of a health information and services delivery system for young people in this area, namely: what services are currently available and what are any barriers to or facilitators of, their provision, experienced by healthcare workers. Additional themes emerged from the data. Thematic analysis of the interview transcripts was conducted and data saturation was reached [55]. Initial coding of interview transcripts was conducted and themes were then visually mapped, with the inclusion of quotes, to provide a detailed picture of the information pertaining to each theme that emerged from the eight interviews. A second reviewer reviewed the results of the thematic analysis alongside the original transcripts, and any discrepancies were resolved by consensus. There was only one discrepancy where a quote had not been included in a relevant thematic map and this was resolved by its inclusion.

This study was approved by the Human Research Ethics Committee (Medical) of The University of the Witwatersrand (Number: M110360). Permission to work with the clinics was granted by the relevant provincial, district and sub-district health authorities. Informed consent was obtained from all interviewees. All eight professional nurses (all female), representing the eight different health facilities, agreed to participate.

## Results

The results are presented to address each objective in turn. Quotes are attributed to clinics using a number to protect the confidentiality of the respondents.

### Services provided at the health facilities

All health facilities were open seven days a week; the seven clinics from 7 am- 4 pm and the health centre 24 hours a day. All facilities provided family planning, treatment for minor ailments, HIV testing and counselling, pregnancy testing, antenatal care, health education and, since April 2011, antiretroviral treatment. Termination of pregnancies (TOP) up to 12 weeks was offered at the health centre. After 12 weeks gestation, pregnant women requesting terminations were referred to local hospitals (between 25 and 45 km from the health centre). The health centre and six of the seven clinics provided laboratory testing for STIs, and the remaining clinic conducted vaginal examinations for syndromic STI diagnosis. Legally adolescents can access health services, including TOP, HIV testing and contraceptives, without parental consent from 12 years of age in South Africa [56]. However, half of interviewees at the clinics in this area reported requiring those under 14 years (and in one case, under 18 years) to come with an adult.

#### Provision of the youth friendly services programme

Participants’ perceptions were that only two facilities had ever provided YFS and that only one was currently providing these services. At both of these facilities the training they received took place before 2006, when the DoH took over the programme’s management. The nurse interviewed at one of these facilities reported that the clinic had previously had an assessment as part of the NAFCI programme and had achieved >90% of the NAFCI “adolescent-friendly” standards. However, the nurse interviewed could not remember when this assessment took place and reported that this facility was no longer providing the YFS programme.

Among the facilities that had never provided YFS, there was enthusiasm to deliver youth-friendly health services. One nurse said: *“My wish, I was… wishing to have that place for youth. To give them enough time and a special person who will work with them, not someone who is working with this, working with this, working with this… I think this will help a lot, but it needs a person with skills, who has trained a lot” (Clinic 3)*. Another said: “*We are interested to have Youth Friendly Services but we don’t have. I don’t know why. Maybe it is because of the poor infrastructure so there is no place for Youth Friendly Services, no place just for teenagers.” (Clinic 7).* Four of the eight facilities had groundBREAKERS (volunteer peer educators aged 18-25 years) trained and supported by loveLife to promote safe sexual behaviour and HIV prevention by engaging with young people in schools, clinics, youth centres and youth groups [36]. This included the two facilities that had provided YFS.

#### Barriers to and facilitators of the provision of youth-friendly health services

##### Human resource issues

The most common barriers to providing health services to young people, and to providing YFS specifically that were reported related to shortages of staff who had received training on the provision of youth-friendly health services and the lack of a dedicated space for young people at the facilities. At the one facility providing YFS two nurses had been trained. At the second facility that had previously provided YFS, it was reported that one nurse had been trained but had since died and that this facility was no longer implementing the programme. These facilities identified shortages of trained staff as a barrier to providing YFS. Four of the facilities that had never implemented YFS identified that having “their own person” who would “work with them” and who has “trained a lot” would help them to provide health services to young people at the clinic and in schools.

At one facility the nurse interviewed said: “We need a special person. Especially the youth, if they know ‘I’m going there, I’m going there to find Sister Anita* (*not her real name)*, I’m going to divulge my problems, my everything, my what what what what what what.’”* (*Clinic 3).* At another the nurse suggested: *“When the adolescent come today they see this one, tomorrow they see that one, they are not happy… Because maybe they trust you and then tomorrow they don’t get you.” (Clinic 6).*

All clinics reported maintaining confidentiality for young people, however, breaches to parents emerged in the narratives at two facilities. At one facility in response to a question on whether the clinic provided pregnancy tests to young people the nurse reported: *“Of course, we do that. With the mothers’ permission, of course, because most of them are being brought in by the mothers… You know sometimes, the mothers they query the child when the child gets sick and they say uh-huh, let me take the child. They know their lifestyle, of course some of the children they are very naughty, you find that the child is not there in the family maybe during the night and you find that the mother is worried… So immediately the mother comes with the child and tells us that, “no, this child, I think she is sexually active now, may you please do something?” And then we start counselling the mother together with the child, and we explain to the child that this is the procedure that we are going to do, we are going to check for pregnancy, we are going to test you, as your mother gave us the permission to see if you are not actually (HIV) positive, you are not pregnant, and then after that we give the results to the mother.” (Clinic 2).* This quote reveals a lack of confidentiality from parents at this clinic and suggests limited choice on behalf of the young person to refuse an HIV or pregnancy test if their caregiver requests it. Judgmental attitudes in relation to young people’s sexual activity are evident; these are branded as *“naughty”*.

##### Infrastructural issues

The idea that young people “need their own (dedicated) space” at the clinic was voiced at five facilities and one of the three facilities that did not mention this already had a space for YFS. One of the facilities that raised this issue had previously provided YFS and had a separate “Adolescent Health Clinic” building. When asked how the provision of health services to young people could be facilitated at this clinic, the nurse interviewed suggested: *“Maybe they have built another house where there will be space. Because sometimes you find that we are using this (the adolescent clinic building) to see even the adult patients, like the chronic and those who are getting ART. So it is a problem, because they are supposed to queue with everybody here. But if the structure is changed, the infrastructure is changed and they have got their own thing maybe it will motivate them… If we had the structure everything would be fine because they would go there and ask the staff.”* (*Clinic 6).*

This idea was reiterated at other health facilities: *“What I think would be the most helpful for us is for us to have enough space here, so that we can have a place to educate our youth, especially the youth-friendly services, I like them the most but we don’t have the space. That’s the problem. If we can have the space or more rooms or maybe a Zozo (temporary structure) or something like that so that we can attend them fully, not partially.” (Clinic 4).* The idea of a dedicated space and a dedicated person as potential facilitators to the provision of health services for young people overlapped. One nurse suggested*: “if they could have their own place and their own person to attend them they would feel free and more would come”. (Clinic 7)*. In response to a question about how health services for young people at the facility could be improved another said: *“Maybe if we can have a space, create a space in the clinic, and say this is for the youth, and we have somebody who is well trained to handle the youth.” (Clinic 8).* At three facilities, in addition to lack of a dedicated space, a lack of clean, piped water was reported. One facility reported that the lack of weekend laboratory services made it difficult to provide services to pregnant school students.

These findings demonstrate that scale-up of the Youth Friendly Services programme in this sub-district is limited and below Department of Health targets. The main barriers to the provision of health services to young people reported by healthcare workers were the lack of trained staff and the lack of a dedicated space for young people. In addition, at half of the clinics, the right of adolescents from 12 years of age to legally access health services, including TOP, HIV testing and treatment and contraceptives, without parental consent did not appear to be being upheld [56].

## Discussion

The YFS programme has the potential to improve health services for young people, and to improve their health outcomes, and has previously been identified as a successful model of how to implement a youth-friendly clinic programme in terms of the achievement of standards relating to clinic policies and the clinic environment [38,44]. However, although the national DoH target is for 70% of primary healthcare facilities to be implementing the YFS programme by 2012/13 [43], in this rural area, participants perceived scale-up and maintenance by 2011 to have been limited; only one of the eight publicly-funded primary healthcare facilities was reported to be providing YFS. This raises questions about the provision and sustainability of the YFS programme as it is currently implemented in this area.

Two main barriers were reported to the provision of youth-friendly health services. All interviewees identified lack of staff training on how to provide youth-friendly health services and five of the eight suggested that young people need a dedicated space at the clinic, as reported by other studies [57,58]. All health facilities reported providing health services to young people and maintaining confidentiality, however, at half of the clinics, the right of adolescents to legally access health services without parental consent from 12 years of age was not being upheld and breaches of confidentiality to parents were reported in two interviews [56].

Whilst a lack of space may affect service delivery for all ages by limiting privacy, these facilities and many rural (and some urban) health facilities in South (and sub-Saharan) Africa are small and have a relatively low patient throughput. The need for a separate “youth space” is therefore unlikely to be justifiable, feasible or necessary. However, lack of clean, piped water, reported at three facilities, should be addressed to facilitate the provision of hygienic health services.

In a study of NAFCI, two of the areas where clinics performed worst at baseline were: staff training on client-centred care (particularly in relation to adolescents and including values clarification) and knowledge of and policies supporting, the sexual and reproductive health rights of adolescents [45]. However, clinics that implemented NAFCI then performed significantly better in these areas than control clinics [44]. Eight years on, with the programme under DoH management, this study found that lack of youth-friendly training, reported by interviewees and reflected by confidentiality breaches, is again a problem, both in facilities that do and do not report providing YFS. Future training should emphasise the legal right of young people to access health service independently from the age of 12 years, and to receive confidential health services.

The finding that facilities where some staff had received training in Youth Friendly Services still identified limited numbers of trained staff as a barrier to implementing this programme, indicates a need to train more, or ideally all, healthcare workers in each facility. This is supported by evidence from other evaluations of youth-friendly services interventions in South Africa and Tanzania [47,58]. A key issue will be providing this training to all healthcare workers given resource constraints, particularly in low- and middle-income countries. Efforts will need to include the provision of training for all existing, as well as new, healthcare workers, as far as possible. However, to promote sustainability this training should also be incorporated into curricula for basic healthcare worker training.

There is also a need for evidence on the long-term impacts of youth-friendly health services training on the knowledge, attitudes and behaviours of healthcare workers and non-clinical staff to give an indication of how often training should be refreshed to maintain any improvements. Findings from the scale-up of a youth-friendly health services intervention in Tanzania showed that statistically significant improvements in healthcare workers knowledge and attitudes were possible after training conducted as part of the intervention package, evaluated in a cluster-randomised controlled trial, and encouragingly, after training implemented through the district health system as part of the scale-up of this intervention [58]. Over time increasing the coverage of youth-friendly health services training, and the experience of those providing the training, should increase capacity within the public health system which may have additional benefits beyond the Youth Friendly Services programme [58].

Enthusiasm for providing YFS expressed by the professional nurses interviewed is promising for further YFS scale-up, and the development of other interventions to improve young people’s health in this area. Efforts to strengthen the existing, district-based primary healthcare system in order to provide integrated care, including preventive as well as curative services, may require a dramatic shift in the structure of the health service and the broader socio-political environment [59]. These issues must be tackled if the South African DoH is to reach its target of 70% of primary healthcare facilities implementing YFS, and to have a significant and measureable impact on the health of the country’s young people [43].

### Strengths and limitations

This work is limited by the relatively small sample of respondents and the focus on one geographical area. Issues of perceived barriers to the provision of youth-friendly health services were investigated from the perspective of healthcare workers as they are best placed to describe any barriers or facilitators they experience in providing these services, both of which could be useful for the development of a nurse-led, health information and services delivery system for young people in this area. The perspectives of other cadres of clinic staff could also be explored in further work, although the general nature of the barriers and facilitators that emerged from this work are likely to be applicable to other staff. To address important questions relating to young people’s experiences and utilisation (or lack of utilisation) of these services, young people should be involved in research on the design and evaluation of programmes, such as YFS, that aim to improve their health: this will be addressed in further work.

Conducting interviews in English rather than the local language of Shangaan could have been a limitation, however, local fieldworkers from the MRC/Wits Rural Public Health and Health Transitions Research Unit (Agincourt) “Learning, Information dissemination, and Networking with Community” (LINC) office attended interviews to provide cultural and language interpretations where necessary. Finally, the depth of evaluation of the YFS programme itself was limited because just one of the eight health facilities reported providing this programme. Future work could identify successful implementation in other clinics outside this sub-district to identify key learning points that could be applied elsewhere.

## Conclusions

Participants reported that provision of the Youth Friendly Services programme is limited in this sub-district, and below the Department of Health’s target that 70% of primary healthcare facilities should provide these services. Whilst a dedicated “youth space” is unlikely to be feasible or necessary, all facilities have the potential to be youth-friendly in terms of staff attitudes and actions and training should be provided to facilitate this. The importance of training on youth-friendly health services was emphasised by the nurses interviewed, suggesting that provision of such training would be popular among the nurses-in-charge in this area. Based on the barriers to and facilitators of the provision of this programme identified by this work, future training should include an emphasis on young people’s right to receive confidential health services, including the legal right of young people aged 12 years and older to access health service independently in South Africa and the importance of being non-judgmental. More than one member of staff per facility should be trained to allow for staff turnover and to facilitate the maintenance of implementation of the YFS programme.

In 2012 the South African Department of Health released a new National Adolescent and Youth Friendly Health Services Strategy that aims to increase the number of healthcare workers trained to provide the Youth Friendly Services programme. Within each sub-district, a number of YFS demonstration sites will act as training bases for at least three other facilities, which will in turn act as training sites for a further three facilities [60]. Further work will be required to monitor both the success of this cascade model of training provision in terms of the numbers of healthcare workers trained, and the impact of this training on their attitudes and behaviour, and on the impact of this programme.

In addition to their relevance to the South African Department of Health, as one of the few countrywide, government-run youth-friendly clinic programmes in a low- or middle-income country, these results may also be of interest to programme managers and policy makers in other low- and middle-income country settings either implementing or planning to implement youth-friendly health services. However, the delivery of this programme may be subject to cultural and social factors as well as aspects of health system management specific to this setting. These findings highlight that positive policies relating to the provision of youth-friendly health services, such as those that exist in South Africa, should be supported by successful and sustained training to facilitate implementation. Further research may aid this by identifying positive examples of such implementation.

## Abbreviations

AIDS: Acquired immune-deficiency syndrome; DoH: Department of Health; HIV: Human immune-deficiency virus; NAFCI: National Adolescent Friendly Clinic Initiative; NAYFHSS: National Adolescent and Youth Friendly Health Services Strategy; NGO: Non-governmental Organisation; TOP: Termination of pregnancy; WHO: World Health Organisation; YFS: Youth friendly services.

## Competing interests

The authors declare that they have no competing interests.

## Authors’ contributions

RG, KK and SN conceived and designed the study, RG and XG-O designed the interview guides and RG collected the data with the assistance of Kari Riggle. RG, KK, ST and SN conducted the analysis and interpretation. RG wrote the article and all authors critically revised the paper. All authors read and approved the final manuscript.

## Pre-publication history

The pre-publication history for this paper can be accessed here:

http://www.biomedcentral.com/1472-6963/14/259/prepub
